# Factors Predicting Guselkumab Treatment Response in Patients with Moderate-to-Severe Plaque Psoriasis: A Post Hoc Analysis of Korean Real-World Data

**DOI:** 10.3390/jcm15020704

**Published:** 2026-01-15

**Authors:** Young Bok Lee, Bong Seok Shin, Miri Kim, Moo Kyu Suh, Sang Woong Youn, Ji Yeoun Lee, Chul Woo Kim, Ga-Young Lee, Kwang Ho Kim, Jihye An, Youngdoe Kim, Kwang Joong Kim, Dong Hyun Kim, Sang Wook Son

**Affiliations:** 1 Department of Dermatology, Uijeongbu St. Mary’s Hospital, College of Medicine, The Catholic University of Korea, Uijeongbu 11765, Republic of Korea; lyb727@gmail.com; 2 Department of Dermatology, Chosun University College of Medicine, Gwangju 61453, Republic of Korea; derm75@chosun.ac.kr; 3 Department of Dermatology, Yeouido St. Mary’s Hospital, College of Medicine, The Catholic University of Korea, Seoul 07345, Republic of Korea; gimmil@naver.com; 4 Department of Dermatology, Dongguk University College of Medicine, Gyeongju 38067, Republic of Korea; mksuhmd@hanmail.net; 5 Department of Dermatology, Seoul National University College of Medicine, Seoul National University Bundang Hospital, Seongnam 13620, Republic of Korea; swyoun@snu.ac.kr; 6 Department of Dermatology, College of Medicine, Chungbuk National University, Cheongju 28644, Republic of Korea; jyl@chungbuk.ac.kr; 7 Department of Dermatology, Kangdong Sacred Heart Hospital, Hallym University College of Medicine, Seoul 05355, Republic of Korea; kimcw@hallym.ac.kr; 8 Department of Dermatology, Kangbuk Samsung Hospital, Sungkyunkwan University School of Medicine, Seoul 03181, Republic of Korea; gygy.lee@samsung.com; 9 Department of Dermatology, Hallym University Sacred Heart Hospital, Hallym University College of Medicine, Anyang 14068, Republic of Korea; dermakkh@naver.com (K.H.K.); kkj51818@hallym.or.kr (K.J.K.); 10 Medical Affairs, Johnson & Johnson, Seoul 04386, Republic of Korea; jan1@its.jnj.com (J.A.); ykim92@its.jnj.com (Y.K.); 11 Department of Dermatology, CHA Bundang Medical Center, CHA University School of Medicine, Seongnam 13496, Republic of Korea; terios92@hanmail.net; 12 Department of Dermatology, Korea University Ansan Hospital, 516, Gojan 1-dong, Danwon-gu, Ansan 15355, Republic of Korea

**Keywords:** guselkumab, Korea, monoclonal antibody, psoriasis, observational study

## Abstract

**Background:** This study aimed to identify the baseline characteristics predictive of Psoriasis Area and Sensitivity Index (PASI) 90 response to guselkumab and assess treatment effectiveness outcomes for PASI 90 responders and PASI 90 non-responders. **Methods:** This post hoc analysis used data from a prospective, multicenter, observational study of guselkumab in Korean patients with moderate-to-severe psoriasis conducted between February 2019 and March 2022. Stepwise logistic regression analysis was used to identify baseline characteristics predictive of PASI 90 response. **Results:** Of 339 patients, 245 (72.3%) week-28 PASI 90 responders and 94 (27.7%) non-responders were identified. Baseline characteristics significantly predictive of PASI 90 response in multivariate logistic regression were absence of family history of psoriasis (odds ratio [OR]: 0.35; *p* = 0.0266), higher PASI score (OR: 1.22; *p* = 0.0006), higher body surface area of psoriasis involvement (OR: 0.95; *p* = 0.0127), prior phototherapy use (OR: 2.44; *p* = 0.0108), and reduced concomitant topical agent use (OR: 0.41; *p* = 0.0044). More PASI 90 responders versus non-responders achieved absolute PASI score ≤ 2 by week 44 (95.8% vs. 67.5%) and Dermatology Life Quality Index scores of 0 or 1 by week 28 (72.2% vs. 34.0%). **Conclusions:** Guselkumab PASI 90 responders had unique baseline characteristics that may predict positive treatment outcomes.

## 1. Introduction

Plaque psoriasis is a chronic immune-mediated disease that causes excessive keratinocyte proliferation, resulting in erythematous, pruritic, and/or painful scaly skin lesions that can significantly impact patient quality of life (QoL) [1,2,3,4,5]. The introduction of biologic agents that target the aberrant proinflammatory cytokine signaling pathways driving the clinical features of plaque psoriasis has improved both skin clearance and QoL outcomes for patients [6,7,8,9,10,11].

Guselkumab (TREMFYA^®^) is a fully human immunoglobulin G1 monoclonal antibody that binds the p19 subunit of interleukin-23 (IL-23). Guselkumab-mediated inactivation of the IL-23 receptor inhibits the downstream signaling pathways and expansion and survival of T helper 17 cells and other immune cells that express IL-23 receptor; consequently, release of proinflammatory cytokines that promote inflammation and keratinocyte proliferation is suppressed [7,8,12]. Additionally, the native Fc region of guselkumab binds to CD64, a receptor expressed on IL-23-producing myeloid cells, and thereby guselkumab neutralizes IL-23 at the source of production and removes IL-23 from the inflamed tissue microenvironment [13,14]. Two multicenter phase 3 trials (VOYAGE 1 and VOYAGE 2) in moderate-to-severe psoriasis demonstrated that guselkumab was well tolerated, had superior efficacy versus adalimumab, and significantly improved patient-reported outcomes, including QoL [7,8]. The VOYAGE studies supported regulatory approval of guselkumab for the treatment of moderate-to-severe plaque psoriasis in multiple countries across North America, Europe, and Asia [15,16,17,18].

While most patients treated with guselkumab demonstrate substantial improvements in Psoriasis Area and Severity Index (PASI) score from baseline with long-term therapy, a proportion of individuals achieve ≥90% improvement in symptoms (PASI 90 response) at week 28 of guselkumab treatment [2,19,20]. Identification of baseline demographics or clinical characteristics that are associated with PASI 90 response to guselkumab may be informative for therapeutic decision-making and allow healthcare professionals to tailor treatment strategies for specific patients [2]. In addition, the overall prevalence of plaque psoriasis in Asian populations is lower than that in European or North American populations [21], suggesting that Asian patients may have unique genetic, demographic, or clinical characteristics driving disease pathogenesis [22], which may impact response to guselkumab treatment.

A recent post-marketing surveillance study evaluating the safety and efficacy of guselkumab in Korean patients with moderate-to-severe plaque psoriasis offers an overview of the effectiveness of guselkumab in Asian patients in a representative real-world setting [23]. This post hoc analysis aimed to identify baseline characteristics predictive of PASI 90 response to guselkumab and assess treatment effectiveness outcomes for PASI 90 responder and PASI 90 non-responder cohorts.

## 2. Materials and Methods

### 2.1. Study Design and Patients

A prospective, multicenter, observational, post-marketing surveillance study was conducted at 44 clinical centers in South Korea between 25 February 2019 and 25 March 2022, to assess the safety and efficacy of guselkumab in patients with moderate-to-severe plaque psoriasis [23]. Eligible patients were those who met requirements for biologic treatment of plaque psoriasis in South Korea and received guselkumab according to the product label during routine clinical practice [16]. Participating centers enrolled patients consecutively during routine clinic visits to minimize selection bias. Guselkumab was administered by subcutaneous injection at the recommended dose of 100 mg at weeks 0 and 4, followed by 100 mg every 8 weeks thereafter [16]. Patients were followed through 44 weeks for evaluation of treatment effectiveness. Here, we report results from a post hoc comparative analysis.

### 2.2. Data Sources and Data Collection

The primary data sources were the medical records for each individual participating in the study. All data were entered into case report forms. The frequency and timing of patient clinical assessments were in accordance with clinical guidelines. Demographic and clinical characteristics, including disease history, presence of comorbidities, and prior treatments, were collected at baseline (week 0). Guselkumab administration status, concomitant medications, measures of treatment effectiveness (PASI score, body surface area [BSA], Investigator’s Global Assessment [IGA] score), and patient-reported outcome measures (Dermatology Life Quality Index [DLQI]) were collected at baseline (week 0), week 4, and approximately every 8 weeks thereafter (weeks 12–44).

As this was a real-world study, treatment administration and patient visits may not have occurred precisely at every 4- or 8-week interval as per guselkumab label guidance. Despite this, most patients received treatment and were clinically assessed by healthcare professionals within ±2 weeks of the label-defined treatment intervals.

### 2.3. Post Hoc Study Population and Definitions

In this post hoc analysis, patients with PASI scores recorded at baseline and week 28 were selected from the overall study population (*N* = 339). Two patient cohorts were identified: guselkumab PASI 90 responders and PASI 90 non-responders, based on assessment at week 28 of treatment.

### 2.4. Statistical Analyses

This study analyzed all collected variables using descriptive statistics, without a specific hypothesis. Descriptive statistics for continuous variables are presented as mean with standard deviation or median values, and categorical variables are presented as percentage of occurrence within the patient sample. Baseline demographics (patient sex, age at study entry, and body mass index [BMI]), medical history including comorbidities (smoker status; alcohol use; and presence of diabetes mellitus, cardiovascular disease, impaired glucose regulation, hypertension, hyperlipidemia, autoimmune thyroiditis/hypothyroidism/hyperthyroidism, inflammatory bowel disease, autoimmune rheumatic disease, uveitis, malignancy, non-alcoholic fatty liver disease, psychiatric disorders, and palmoplantar pustulosis), and disease characteristics (family history of psoriasis, disease duration, PASI score, total BSA and body areas impacted, IGA score, presence of psoriatic arthropathy, number and type of prior treatments, and concomitant treatments) were compared between the PASI 90 responder and PASI 90 non-responder cohorts. Treatment effectiveness outcomes over the 44-week study period, including the proportion of patients achieving an absolute PASI score ≤ 2 (aPASI2), a DLQI score of 0 or 1 (DLQI 0/1), PASI 75, PASI 90, PASI 100, and an absolute PASI score ≤ 1 (aPASI1), were compared between the PASI 90 responder and PASI 90 non-responder cohorts. Additionally, the time to absolute PASI score of 0 (aPASI0) was analyzed by Kaplan–Meier for both guselkumab PASI 90 responder and PASI 90 non-responder cohorts.

In an exploratory statistical comparison, continuous variables were analyzed, where appropriate, using the Wilcoxon rank sum and *t*-test, while categorical variables were assessed using Pearson’s chi-square test and Fisher’s exact test. Imputation of missing data was not performed. In addition, logistic regression analysis was conducted to investigate potential baseline variables affecting achievement of PASI 90 at week 28. Variables with a *p*-value < 0.1 from the univariate logistic regression analysis were included in the multivariate logistic regression analysis. For exploratory purposes, all statistical analyses were performed using 2-sided tests and results with *p*-values < 0.05 were considered statistically significant. All analyses were performed using the statistical software package SAS 9.4 (Statistical Analysis System, SAS-Institute, Cary, NC, USA).

### 2.5. Ethical Considerations

This observational study was conducted in accordance with the International Society for Pharmacoepidemiology Guidelines for Good Pharmacoepidemiology Practices. The protocol and patient informed consent form were approved by the local institutional review board at all participating clinical sites (Supplemental Table S1). All patients provided written informed consent before participation in the study and could withdraw at any time.

## 3. Results

### 3.1. Patients

A total of 339 patients with moderate-to-severe plaque psoriasis treated with guselkumab were included in this post hoc analysis. Among them, 245 (72.3%) were PASI 90 responders, and 94 (27.7%) were PASI 90 non-responders at week 28. The median duration of guselkumab treatment was 316 days for both cohorts.

### 3.2. Baseline Demographics, Medical History, and Disease Characteristics

Baseline demographics and medical history for guselkumab PASI 90 responders and PASI 90 non-responders are shown in Table 1. PASI 90 non-responders were slightly older than PASI 90 responders (44.5 years vs. 42.0 years); however, this difference was not statistically significant. While median BMI did not significantly differ between cohorts, PASI 90 non-responders had a marginally higher BMI at baseline (26.2 kg/m^2^ vs. 25.3 kg/m^2^). A significant difference was observed between cohorts for smoking status, with a higher proportion of PASI 90 responders reporting that they had never smoked (*p* = 0.042). Alcohol use and other baseline characteristics were comparable between PASI 90 responders and PASI 90 non-responders.

PASI 90 non-responders presented with more comorbid conditions at baseline than PASI 90 responders (Table 1). Notably, the prevalence of diabetes mellitus (10.6% vs. 4.1%, *p* = 0.0218) and hypertension (17.0% vs. 8.6%, *p* = 0.0255) was significantly higher among PASI 90 non-responders. Although not statistically significant, PASI 90 non-responders also had higher incidences of cardiovascular disease (2.1% vs. 0.8%, *p* = 0.3081), rheumatic autoimmune disease (12.8% vs. 9.4%, *p* = 0.3601), non-alcoholic fatty liver disease (1.1% vs. 0%, *p* = 0.2773), and psychiatric disorders (3.2% vs. 0.4%, *p* = 0.0664) than PASI 90 responders. The incidence of other comorbidities was not significantly different between the cohorts.

Significant differences in several baseline disease characteristics were noted between cohorts (Table 2). Compared with PASI 90 non-responders, PASI 90 responders had significantly shorter median disease duration (85.3 months vs. 125.4 months; *p* = 0.0162) and were significantly more likely to have no family history of plaque psoriasis (93.3% vs. 84.9%, *p* = 0.0182). Interestingly, PASI 90 responders exhibited significantly higher mean baseline PASI scores (16.7 vs. 13.8, *p* < 0.0001) and greater total BSA of psoriasis involvement (22.4% vs. 19.3%, *p* = 0.0108) compared with PASI 90 non-responders. Based on IGA score, a higher proportion of PASI 90 responders had moderate disease at baseline than PASI 90 non-responders (59.5% vs. 31.4%, *p* = 0.0034).

Regarding prior treatments, PASI 90 responders had higher usage rates of topical agents, phototherapy, and systemic oral agents than PASI 90 non-responders. Significant differences were noted for topical agents (67.3% vs. 50.0%, *p* = 0.0031) and methotrexate (50.2% vs. 36.2%, *p* = 0.0204). Although not statistically significant, a lower proportion of PASI 90 responders had previous exposure to biologics (16.7% vs. 20.2%, *p* = 0.4526) compared with PASI 90 non-responders. Concomitant treatment use was generally similar between cohorts; however, significantly fewer PASI 90 responders reported concomitant use of topical agents during guselkumab treatment (36.3% vs. 66.0%, *p* < 0.0001).

### 3.3. Predictors of PASI 90 Response to Guselkumab at Week 28

The demographic, medical history, and disease characteristics found to be significant in the univariate analysis (such as female sex; age; smoking status; family history of psoriasis; psoriasis disease duration; psoriasis of the back of the foot; comorbidities including presence of diabetes mellitus, hypertension, and psychiatric disorders; as well as baseline PASI and BSA scores; prior phototherapy or topical agent use; and concomitant topical agent use) were included in the multivariate logistic regression analysis (Table 3). In the multivariate analysis, significant predictors of PASI 90 response to guselkumab included absence of family history of psoriasis (odds ratio [OR]: 0.35; 95% confidence interval [95% CI]: 0.135–0.884; *p* = 0.0266), higher baseline PASI score (OR: 1.22; 95% CI: 1.089–1.367; *p* = 0.0006), higher baseline BSA of psoriasis involvement (OR: 0.95; 95% CI: 0.908–0.989; *p* = 0.0127), prior phototherapy use (OR: 2.44; 95% CI: 1.229–4.858; *p* = 0.0108), and reduced concomitant topical agent use (OR: 0.41; 95% CI: 0.222–0.756; *p* = 0.0044) (Table 3).

### 3.4. Guselkumab Treatment Effectiveness Outcomes

**Figure 1:**
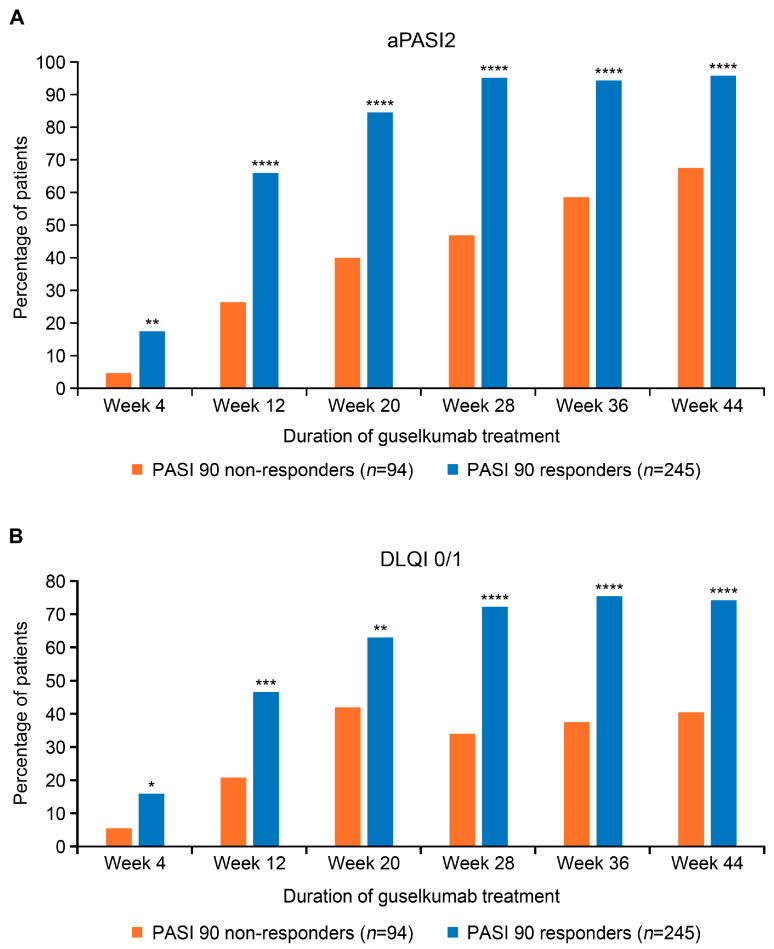
Proportion of guselkumab PASI 90 responders and PASI 90 non-responders achieving aPASI2 (**A**) and DLQI 0/1 (**B**) across the duration of treatment. PASI score of 0 indicates no psoriasis; PASI score > 10 is consistent with severe psoriasis. Lower DLQI scores (0–1) represent no impact on patient’s life; high scores (21–30) represent an extremely large impact on patient’s life. * Denotes *p*-value < 0.05; ** denotes *p*-value < 0.01; *** denotes *p*-value < 0.001; **** denotes *p*-value < 0.0001. aPASI2, absolute Psoriasis Activity Severity Index score of ≤2; DLQI 0/1, Dermatology Life Quality Index score of 0 or 1; PASI, Psoriasis Area and Severity Index.

**Figure 2:**
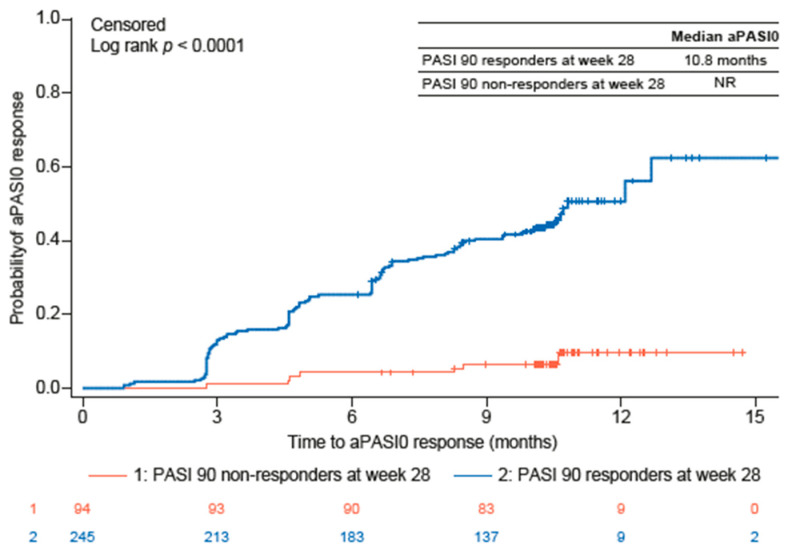
Time to aPASI0 for guselkumab PASI 90 responders and PASI 90 non-responders. aPASI0, absolute Psoriasis Activity Severity Index score of 0; PASI, Psoriasis Area and Severity Index; NR, not reached.

Over the 44-week study period, a significantly higher proportion of guselkumab PASI 90 responders than PASI 90 non-responders also achieved aPASI2 at all timepoints evaluated (Figure 1A). By week 4, 17.3% of PASI 90 responders and 4.6% of PASI 90 non-responders achieved aPASI2. By week 20, 84.5% of PASI 90 responders achieved aPASI2, with nearly all of these responders (95.8%) reaching aPASI2 by week 44. In contrast, only 40.0% of PASI 90 non-responders achieved aPASI2 by week 20, increasing to 67.5% by week 44. Similarly, a significantly greater proportion of PASI 90 responders attained DLQI 0/1 at all timepoints (Figure 1B). The difference was particularly notable at week 28 (72.2% vs. 34.0%, *p* < 0.0001), week 36 (75.4% vs. 37.5%, *p* < 0.0001), and week 44 (74.2% vs. 40.5%, *p* = 0.0001). Analyses of PASI 75, PASI 90, PASI 100, and aPASI1 over time showed a similar trend, with a greater proportion of PASI 90 responders achieving response at most timepoints than PASI 90 non-responders (Supplemental Figure S1). The median time to achieve aPASI0 was substantially shorter for PASI 90 responders (10.8 months) compared with PASI 90 non-responders, for whom the median time was not reached (Figure 2). By week 44, 45.7% of PASI 90 responders and 7.4% of PASI 90 non-responders achieved aPASI0.

## 4. Discussion

In this study of Korean patients with moderate-to-severe plaque psoriasis treated with guselkumab in a real-world setting, approximately 72% of patients achieved a PASI 90 response by week 28, indicating a substantial reduction in disease severity. Interestingly, PASI 90 responders were more likely to present with higher baseline PASI and BSA scores. Key baseline characteristics significantly predictive of a PASI 90 response versus a PASI 90 non-response included shorter disease duration and fewer comorbidities, underscoring the importance of early intervention and comprehensive patient assessment to optimize treatment outcomes with guselkumab. Notably, we observed that patients who were PASI 90 responders at week 28 had consistently good outcomes over the 44-week treatment period. Over 45% of PASI 90 responders achieved complete skin clearance (aPASI0) with guselkumab across the duration of treatment. PASI 90 responders maintained their therapeutic gains, with 95.8% achieving an absolute PASI score ≤ 2 by week 44, compared with 67.5% of PASI 90 non-responders. Furthermore, QoL improvements were substantial, with 72.2% of PASI 90 responders attaining a DLQI score of 0 or 1 by week 28, compared with 34.0% of PASI 90 non-responders.

Differences in treatment response observed between patients in this study are not unique to guselkumab and are evident across the spectrum of biologics used to treat psoriasis, with varying patient characteristics reported to be associated with better response depending on the biologic. Reports based on multiple real-world studies have identified factors that may be associated with, or predictive of, response to biologic treatments in patients with psoriasis; however, the variables reported as statistically significant are inconsistent between studies, likely due to differences in study design, patient populations, and treatment strategies. In line with the data presented from this analysis, several reports indicate that higher baseline PASI scores may be associated with a good response to biologics [24,25]. Findings from one Chinese study suggested that patients with a super response to adalimumab (those who achieved PASI 100 at week 12 and at either week 24 or 32) had a higher PASI score at baseline than those who achieved a non-super response, although the difference was not significant [24]. Additionally, a study conducted in Denmark indicated that the odds of achieving PASI 90 after biologic treatment for 3 or 6 months were significantly higher in biologic-naïve patients with a higher baseline PASI score [25]. Despite this, and in contrast with the results presented here, the Danish study also reported that those patients who used concomitant local treatment with their biologic had increased odds of achieving PASI 90 after 3 or 6 months of treatment; however, it was unclear what comprised local treatment (i.e., topical agents and/or phototherapy) [25]. Other studies, including the PSO-BIO-REAL study (*n* = 846) and the IMMerge study (*n* = 327), have also reported that biologic-naïve patients were more likely to achieve PASI 90 [26,27]; however, the current study found no significant differences in previous biologic use between PASI 90 responders and PASI 90 non-responders.

Consistent with the data presented here, many real-world studies investigating the variables associated with treatment response to biologics targeting IL-17 and IL-23 suggest limited associations between patient sex, BMI, or previous biologic exposure and achievement of PASI 90 (though, as noted previously, some studies did find that biologic-naïve patients were more likely to achieve PASI 90 response) [27,28,29,30,31,32]. Several studies have reported that patients without obesity or diabetes mellitus at baseline generally have a better treatment response [33]. It has been suggested that this may be due to patients having an overall lower systemic inflammatory burden at baseline; however, further studies are required to confirm this hypothesis. While the current study indicates that a significantly lower proportion of PASI 90 responders had diabetes mellitus at baseline versus PASI 90 non-responders, this was not found to be a significant predictor of response to guselkumab in the multivariate logistic regression analysis. Similarly, hypertension and several other baseline characteristics showed association with PASI 90 response in the univariate logistic regression but were not significant in the multivariate logistic regression. These variables likely represent potential confounders that had been adjusted for in the multivariate analysis.

Another factor that may represent a robust significant predictor of PASI 90 response to biologics is the absence of nail psoriasis [34,35,36]. Data from previous studies indicate that patients without nail psoriasis who are treated with biologics are twice as likely to achieve complete skin clearance after 12 months of treatment than those with psoriatic nail involvement [35]. Together with nail psoriasis, the absence of hypertension at baseline was also identified as a significant predictor of good treatment response to biologics [35]. In the current study, lower proportions of PASI 90 responders had nail involvement (18.8% vs. 24.5%, respectively) and hypertension (8.6% vs. 17.0%, respectively) than PASI 90 non-responders at baseline, suggesting a similar trend with other studies, although this was not a significant predictor of guselkumab response. Interestingly, patients who present with nail psoriasis also typically have a longer disease duration than those without nail involvement [34,36]. Of note, findings from our real-world study indicate that PASI 90 responders had significantly shorter disease duration at baseline than PASI 90 non-responders. These data are consistent with the GUIDE study, which reported that patients with short disease duration were more likely to be guselkumab PASI 100 responders (defined as achievement of 100% improvement in PASI score at both weeks 20 and 28 of guselkumab treatment) than those with long disease duration [36].

The real-world findings presented here differ from a post hoc analysis of pooled patient data from the VOYAGE 1 and 2 randomized controlled studies. The VOYAGE studies demonstrated that baseline factors significantly associated with guselkumab PASI 100 response were younger age, less severe disease (including the absence of nail psoriasis), lower BMI, and lower PASI and IGA scores [2]. None of these factors were significant predictors of PASI 90 response in the current study. Notably, the current study suggests that a higher baseline PASI score, rather than a lower baseline PASI score, is predictive of PASI 90 response. The differences in the results between these studies may be due to several factors. While both were post hoc analyses, the current study used data from a real-world clinical setting in Korea whereas the VOYAGE 1 and 2 studies utilized clinical trial data from a patient population subjected to strict inclusion and exclusion criteria. Additionally, patient ethnicity may represent a confounding variable for factors that may significantly predict super response; the majority (~85%) of patients in the VOYAGE trials were White [2], whereas all participants in the current study were Asian. Furthermore, the post hoc analysis of the VOYAGE studies evaluated a cohort comprised specifically of PASI 100 responders, whereas the current study analyzed patients categorized as PASI 90 responders, with PASI 100 responders included in this broader cohort. Patients who achieve PASI 100 response to guselkumab constitute a discrete population of patients and, as such, may have unique baseline characteristics that influence treatment response.

The current study has several limitations that require consideration. The non-randomized observational design of the study, with no comparator or control arm, limits the robustness of the evidence. This was a post hoc analysis of an observational study and was not specifically designed for statistical comparisons between PASI 90 responder and PASI 90 non-responder patient cohorts. Given the exploratory nature of this post hoc analysis, the results are intended to describe observed associations rather than to support formal hypothesis testing. Despite utilizing data from 44 clinical centers, the overall sample size was small, and the majority of patients included in this analysis were male. Patients with PASI scores recorded at baseline and week 28 were included, which may have resulted in selection bias towards patients who stayed well and remained on the study. Further studies using larger patient cohorts are required to examine and validate factors that may impact guselkumab treatment response. In addition, due to the observational nature of the study, data were missing for certain variables such as BMI, DLQI, and IGA in a proportion of patients, which might have had an impact on the analysis results. The results generated from the stepwise logistic regression should be interpreted as hypothesis-generating, which warrant further investigation in prospective clinical studies. No causal relationship could be made based on logistic regression analysis, and certain predictors for PASI 90 response, such prior phototherapy use and reduced concomitant topical agent use, might reflect treatment patterns in responders versus non-responders.

Identification of patients who are likely to achieve PASI 90 response to guselkumab treatment, as well as those who may prove to be non-responders, may allow for optimization of treatment strategies and improve long-term patient outcomes, including complete skin clearance. The typical trial and error approach to determine whether individual patients may achieve a desirable response to biologic treatments has been shown to impact patient QoL. A recent study from Korea indicated that up to 21.6% of patients with moderate-to-severe plaque psoriasis switched from their initial biologic agent to a second biologic agent due to a lack of efficacy [37]; however, treatment switching in an attempt to improve therapeutic response was reported to pose a significant burden for patients [38]. Additionally, patients treated with therapies that demonstrate limited efficacy in terms of PASI response report poorer DLQI and mental health scores than those who achieve complete skin clearance [39,40]. These data underscore the importance of early and effective treatment interventions that are tailored to the individual patient to improve both long-term clinical and QoL outcomes.

## 5. Conclusions

In conclusion, data from this post-marketing study of patients who received guselkumab for the treatment of moderate-to-severe plaque psoriasis in Korea indicate that PASI 90 responders to guselkumab have unique baseline characteristics that may predict treatment efficacy. Disease duration was significantly shorter for PASI 90 responders compared with PASI 90 non-responders, suggesting an association between early intervention and better treatment outcomes. Guselkumab treatment effectiveness outcomes were consistently good for PASI 90 responders, with most reporting substantial improvements in QoL over the 44-week treatment period. The baseline characteristics predictive of response to guselkumab identified in this study are hypothesis-generating, warranting confirmation in future studies, which may optimize treatment decision-making and facilitate selection of tailored treatment strategies to improve skin clearance outcomes.

## Figures and Tables

**Table 1:** Baseline demographics and medical history of PASI 90 responders and PASI 90 non-responders.

	PASI 90 Responders (*n* = 245)	PASI 90 Non-Responders (*n* = 94)	*p*-Value
Sex, *n* (%)			
Male	166 (67.8)	65 (69.1)	0.8052
Female	79 (32.2)	29 (30.9)	
Age, years	42.0	44.5	0.0712
BMI			
* n*	123	47	
BMI (kg/m^2^)	25.3	26.2	0.1909
Smoking status, *n* (%)			
* n*	220	75	
Non-smoker	132 (60.0)	41 (54.7)	**0.0402**
Past smoker	18 (8.2)	14 (18.7)	
Current smoker	70 (31.8)	20 (26.7)	
Alcohol use, *n* (%)			
* n*	220	74	
Never used alcohol	72 (32.7)	29 (39.2)	0.1097
Formerly used alcohol	24 (10.9)	13 (17.6)	
Currently using alcohol	124 (56.4)	32 (43.2)	
Comorbidities, *n* (%)			
Autoimmune rheumatic disease	23 (9.4)	12 (12.8)	0.3601
Hyperlipidemia	22 (9.0)	8 (8.5)	0.8917
Hypertension	21 (8.6)	16 (17.0)	**0.0255**
Diabetes mellitus	10 (4.1)	10 (10.6)	**0.0218**
Cardiovascular disease	2 (0.8)	2 (2.1)	0.3081
Impaired glucose regulation	2 (0.8)	0	1.0000
Autoimmune thyroiditis/hypo-/ hyperthyroidism	2 (0.8)	0	1.0000
Psychiatric disorders	1 (0.4)	3 (3.2)	0.0664
Uveitis	1 (0.4)	0	1.0000
Malignancy	1 (0.4)	0	1.0000
Non-alcoholic fatty liver disease	0	1 (1.1)	0.2773
Palmoplantar pustulosis	0	1 (1.1)	0.2773

Patient numbers (*n*) are reported in the headers unless otherwise specified. Median values are reported unless otherwise stated. *p*-values < 0.05 are bolded to denote significance. BMI, body mass index; PASI, Psoriasis Area and Severity Index.

**Table 2:** Baseline disease characteristics of PASI 90 responders and PASI 90 non-responders.

	PASI 90 Responders (*n* = 245)	PASI 90 Non-Responders (*n* = 94)	*p*-Value
Family history of psoriasis, *n* (%)			
* n*	240	86	
Yes	16 (6.7)	13 (15.1)	**0.0182**
Disease duration, months	85.3	125.4	**0.0162**
Psoriasis morphology, *n* (%)			
Scalp	131 (53.5)	43 (45.7)	0.2027
Face	102 (41.6)	42 (44.7)	0.6113
Neck	95 (38.8)	40 (42.6)	0.5247
Chest	159 (64.9)	64 (68.1)	0.5798
Abdomen	193 (78.8)	70 (74.5)	0.3946
Back	190 (77.6)	78 (83.0)	0.2716
Upper arm	197 (80.4)	73 (77.7)	0.5737
Elbow	173 (70.6)	63 (67.0)	0.5199
Lower arm	192 (78.4)	75 (79.8)	0.7748
Palm	23 (9.4)	7 (7.4)	0.5732
Back of hand	56 (22.9)	26 (27.7)	0.3553
Nail	46 (18.8)	23 (24.5)	0.2439
Upper leg	193 (78.8)	77 (81.9)	0.5204
Knee	165 (67.3)	62 (66.0)	0.8076
Lower leg	218 (89.0)	89 (94.7)	0.1080
Foot sole	30 (12.2)	13 (13.8)	0.6947
Back of foot	44 (18.0)	25 (26.6)	0.0771
Buttock	119 (48.6)	49 (52.1)	0.5577
Genitalia	16 (6.5)	9 (9.6)	0.3371
Concurrent psoriatic arthropathy, *n* (%)	20 (8.2)	10 (10.6)	0.4726
PASI score (0–72), mean (SD)	16.7 (6.5)	13.8 (4.9)	**<0.0001**
BSA, mean (SD)	22.4 (15.1)	19.3 (14.6)	**0.0108**
IGA score, *n* (%)			
* n*	148	51	
Minimal	3 (2.0)	1 (2.0)	**0.0034**
Mild	47 (31.8)	27 (52.9)	
Moderate	88 (59.5)	16 (31.4)	
Severe	10 (6.8)	7 (13.7)	
DLQI, *n* (%)			
* n*	152	59	
0–1	6 (3.9)	2 (3.4)	0.3908
2–5	14 (9.2)	3 (5.1)	
6–10	22 (14.5)	15 (25.4)	
11–20	60 (39.5)	22 (37.3)	
21–30	50 (32.9)	17 (28.8)	
Number of prior biologics, *n* (%)			
0	204 (83.3)	75 (79.8)	0.6593
1	35 (14.3)	17 (18.1)	
2	5 (2.0)	1 (1.1)	
≥3	1 (0.4)	1 (1.1)	
Type of prior treatment, *n* (%)			
Topical	165 (67.3)	47 (50.0)	**0.0031**
Phototherapy	179 (73.1)	60 (63.8)	0.0952
Systemic oral agent	226 (92.2)	84 (89.4)	0.3955
Steroid	6 (2.4)	5 (5.3)	0.1859
Methotrexate	123 (50.2)	34 (36.2)	**0.0204**
Cyclosporine	137 (55.9)	59 (62.8)	0.2531
Acitretin	10 (4.1)	5 (5.3)	0.5697
Others	2 (0.8)	5 (5.3)	0.0193
Biologics	41 (16.7)	19 (20.2)	0.4526
Concomitant treatment, *n* (%)	96 (39.2)	63 (67.0)	**<0.0001**
Type of concomitant treatment, *n* (%)			
Systemic oral agent	17 (6.9)	6 (6.4)	0.8555
Phototherapy	2 (0.8)	1 (1.1)	1.0000
Topical	89 (36.3)	62 (66.0)	**<0.0001**

Patient numbers (*n*) are reported in the headers unless otherwise specified. *p*-values < 0.05 are bolded to denote significance. PASI score of 0 indicates no psoriasis; PASI score > 10 consistent with severe psoriasis. DLQI score of 0–1 represents no impact on a patient’s life; high scores (21–30) represent an extremely large impact on a patient’s life. BSA, body surface area; DLQI, Dermatology Life Quality Index; IGA, Investigator’s Global Assessment; PASI, Psoriasis Area and Severity Index; SD, standard deviation.

**Table 3:** Univariate and multivariate logistic regression analyses identifying baseline characteristics predictive of PASI 90 response to guselkumab.

	Univariate Analysis			Multivariate Logistic Regression		
Characteristic	OR	95% CI	*p*-Value	OR	95% CI	*p*-Value
Female sex	1.067	0.639–1.782	0.8053	0.943	0.446–1.991	0.8774
Age, years	0.981	0.964–0.998	0.0302	0.995	0.971–1.019	0.6763
Positive family history of psoriasis	0.401	0.184–0.873	0.0214	0.345	0.135–0.884	**0.0266**
Smoker status						
Current	1.087	0.592–1.997	0.7877	0.974	0.441–2.155	0.9489
Past	0.399	0.183–0.872	0.0213	0.428	0.165–1.109	0.0807
Disease duration, months	0.997	0.995–1.000	0.0173	0.999	0.996–1.002	0.5695
Psoriasis morphology						
Back of foot	0.604	0.344–1.060	0.0787	0.522	0.242–1.127	0.0978
Comorbidities						
Diabetes mellitus	0.358	0.144–0.890	0.0270	0.713	0.195–2.607	0.6092
Hypertension	0.457	0.227–0.920	0.0282	0.754	0.286–1.984	0.5670
Psychiatric disorders	0.124	0.013–1.211	0.0726	0.192	0.015–2.451	0.2039
Baseline PASI score	1.111	1.051–1.175	0.0002	1.220	1.089–1.367	**0.0006**
Baseline BSA score	1.016	0.997–1.034	0.0947	0.948	0.908–0.989	**0.0127**
Prior treatment use						
Topical agents	2.063	1.271–3.348	0.0034	1.464	0.789–2.717	0.2270
Phototherapy	1.537	0.926–2.551	0.0964	2.444	1.229–4.858	**0.0108**
Concomitant treatment use						
Topical agents	0.294	0.179–0.485	<0.0001	0.409	0.222–0.756	**0.0044**

Variables with a *p*-value of <0.1 in the univariate analysis were included in the multivariate logistic regression analysis; age, sex, and baseline PASI score were used as base covariates for the multi-variate logistic regression analysis. *p*-values < 0.05 are bolded to denote significance. BSA, body surface area; CI, confidence interval; OR, odds ratio; PASI, Psoriasis Area and Severity Index.

## Data Availability

The de-identified datasets used and/or analyzed during the current study are available from the corresponding author on reasonable request.

## Supplementary Materials

The following supporting information can be downloaded at: https://www.mdpi.com/article/10.3390/jcm15020704/s1, Table S1: List of institutional review boards (IRBs); Figure S1: Proportion of guselkumab PASI 90 responders and PASI 90 non-responders achieving PASI 75 (A), PASI 90 (B), PASI 100 (C), and aPASI1 (D) across the duration of treatment.

## Abbreviations

The following abbreviations are used in this manuscript:

aPASI	Absolute Psoriasis Area and Sensitivity Index
aPASI0	Absolute Psoriasis Activity Severity Index score of 0
aPASI1	Absolute Psoriasis Activity Severity Index score of ≤1
aPASI2	Absolute Psoriasis Activity Severity Index score of ≤2
BMI	Body mass index
BSA	Body surface area
CI	Confidence interval
DLQI	Dermatology Life Quality Index
DLQI 0/1	Dermatology Life Quality Index score of 0 or 1
IGA	Investigator’s Global Assessment
IL	Interleukin
OR	Odds ratio
PASI	Psoriasis Area and Sensitivity Index
QoL	Quality of life
SD	Standard deviation
