# Supplementary material for: Factors Predicting Guselkumab Treatment Response in Patients with Moderate-to-Severe Plaque Psoriasis: A Post Hoc Analysis of Korean Real-World Data
Source: J Clin Med. 2026 Jan 15;15(2):704. doi: 10.3390/jcm15020704 (PMC12841860; doi:10.3390/jcm15020704)

Supplemental materials

Title:

Factors predicting guselkumab treatment response in patients with moderate-to-severe plaque psoriasis: A post hoc analysis of Korean real-world data

Table of contents

Supplemental Table S1: List of institutional review boards (IRBs). 2

Supplemental Figure S1. Proportion of guselkumab PASI 90 responders and PASI 90 non-responders achieving PASI 75 (A), PASI 90(B), PASI 100 (C), and aPASI1 (D) across the duration of treatment. 7

Supplemental Table S1: List of institutional review boards (IRBs).

IRB Full name	IRB no.	IRB approval date (year-month-day)
Hallym University Kangnam Sacred Heart Hospital Institution Review Board	HKS PMS2019-009	2019-07-16
Hallym University Kangnam Sacred Heart Hospital Institution Review Board	HKS PMS2019-002	2019-03-29
Yonsei University Gangnam Severance Hospital Institutional Review Board	3-2018-0354	2019-02-14
Kyung Hee University Hospital at Gangdong INSTITUTIONAL REVIEW BOARD	KHNMC PMS2019-009	2019-11-26
KangDong Sacred Heart Hospital Institutional Review Board	KANGDONG PMS2019-005	2019-05-20
KangDong Sacred Heart Hospital Institutional Review Board	KANGDONG PMS2019-004	2019-05-20
Kangbuk Samsung Hospital Institutional Review Board	KBSMC PMS2019-008	2019-04-09
KonKuk University Medical Center Institutional Review Board	KUMC IRB PMS1120-126-021	2019-03-21
Kyungpook National University Hospital Institutional Review Board	KNUH IRB PMS2019-008	2019-06-19
Kyung Hee University Hospital Institutional Review Board	KHUH PMS2019-028	2019-07-24
Korea University Ansan Hospital Institution Review Board	2019AS0055	2019-03-12
Korea University Anam Hospital Institution Review Board	2020AN0060	2020-02-17

National Medical Center Institutional Review Board	H-1903-100-001<br>(NMC IRB PMS1903-001-001)	2019-05-24
Dankook University Hospital Institutional Review Board	DKUH IRB PMS2019-006	2019-05-15
Daugu Catholic Univ. Medical Center Institutional Review Board	CR-19-025<br>(DCUMC PMS2019-003)	2019-04-03
Dongguk University Gyeongju Hospital Institutional Review Board	110757-201903-HR-01	2019-05-09
Dongguk University Ilsan Hospital Institutional Review Board	DUIH IRB PMS2019-008	2019-06-20
Hallym University Dongtan Sacred Heart Hospital Institutional Review Board	HDT IRB PMS2019-006	2019-05-28
Boramae Medical Center Institutional Review Board	20190530/30-2019-57/063	2019-05-30
Inje University Busan Paik Hospital Institutional Review Board	BPIRB PMS2019-068	2019-05-27
The Catholic University Of Korea Bucheon ST. Mary's Hospital Institutional Review Board	HC19MODP0078	2019-09-04
Seoul National Univiersity Hospital Institutional Review Board	B-1904-532-202	2019-03-27
CHA Bundang Medical Center Institutional Review Board	CHAMC IRB PMS2019-002	2019-02-18
Samsung Medical Center Institutional Review Board	SMC PMS2019-007	2019-04-03
The Catholic University Of Korea Seoul ST. Mary's Hospital Institutional Review Board	KC20MSDP0375	2020-08-10

The Catholic University Of Korea Seoul ST. Mary's Hospital Institutional Review Board	KC19MSDP0736	2019-10-29
The Catholic University Of Korea ST. Vicent's Hospital Institutional Review Board	VC19MSDP0272	2019-12-12
Soon Chun Hyang University Hospital Bucheon Institutional Review Board	SCHBC PMS2019-005	2019-05-02
Soon Chun Hyang University Hospital Cheonan Institutional Review Board	SCHCA IRB PMS2019-005	2019-05-29
Ajou University Hospital Institutional Review Board	AJIRB-MED-PMS-19-156	2019-05-10
Ajou University Hospital Institutional Review Board	AJIRB-MED-PMS-19-157	2019-05-10
Ajou University Hospital Institutional Review Board	AJIRB-MED-OBS-18-512	2019-02-18
Pusan National University Yangsan Hospital Institutional Review Board	06-2019-021	2019-11-14
The Catholic University Of Korea Yeouido ST. Mary's Hospital Institutional Review Board	SC19MODE0106	2019-09-10
Nowon Eulji Medical Center, Eulji University Institutional Review Board	EMCS PMS2019-004	2019-09-26
The Catholic University Of Korea Uljeongbu ST. Mary's Hospital Institutional Review Board	UC19MODP0102	2019-08-29
Inje University Sanggye Paik Hospital Institutional Review Board	SGPAIK IRB PMS2019-020	2019-09-11

The Catholic University Of Korea Incheon ST. Mary's Hospital Institutional Review Board	OC19MODP0127	2019-09-06
Inha University Hospital Institutional Review Board	PMS2019-021	2019-09-30
Inje University Ilsan Paik Hospital Institutional Review Board	ISPAIK PMS2019-014	2019-05-29
Chonnam National University Hospital Institutional Review Board	CNUH-2019-164	2019-06-08
Chosun University Hospital Institutional Review Board	CHOSUN IRB PMS2019-004	2019-05-07
Chung-Ang University Hospital Institutional Review Board	1908-006-16275	2019-09-19
Chung-Ang University Hospital Institutional Review Board	1908-012-16278	2019-10-13
VHS Medical Center Institutional Review Board	BOHUN PMS2019-009	2019-06-26
Chungnam National University Hospital Institutional Review Board	CNUH PMS2019-019	2019-09-02
Chungbuk National University Hospital Institutional Review Board	CBNUH PMS2019-006	2019-05-23
Hallym University Sacred Heart Hospital Institutional Review Board	HALLYM PMS2019-001	2019-01-22
Hallym University Sacred Heart Hospital Institutional Review Board	HALLYM PMS2019-002	2019-01-29
Hanyang University Seoul Hospital Institutional Review Board	HYUH PMS2019-001	2019-04-05

Inje University Haeundae Paik Hospital Institutional Review	HPIRB PMS2019-005	2019-04-05

Supplemental Figure S1. Proportion of guselkumab PASI 90 responders and PASI 90 non-responders achieving PASI 75 (A), PASI 90(B), PASI 100 (C), and aPASI1 (D) across the duration of treatment.

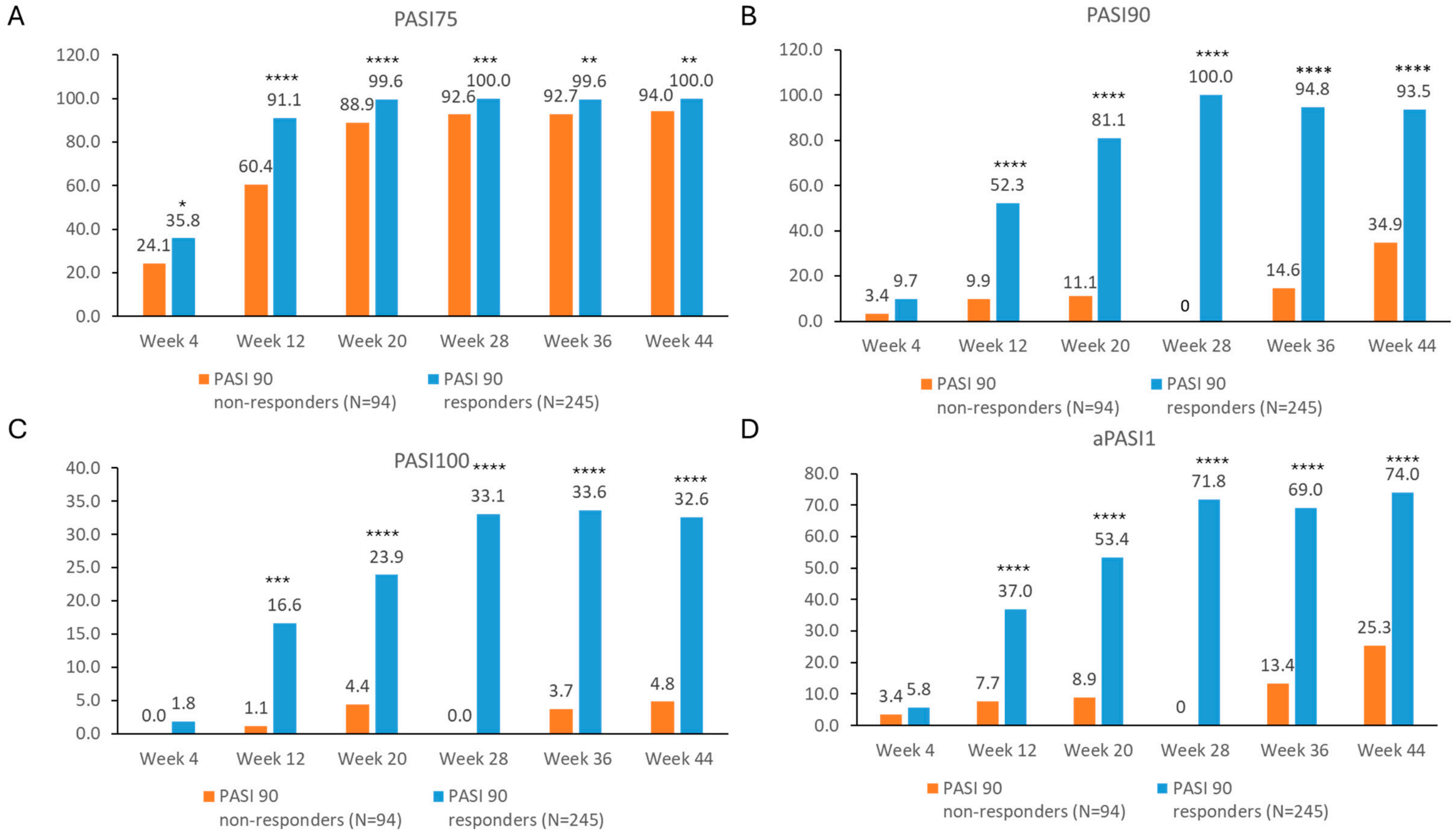

Supplement: Supplementary file 1 [file jcm-15-00704-s001.zip › jcm-4024552-supplementary.pdf]
